# Impact of Pretreatment Ischemic Location on Functional Outcome after Thrombectomy

**DOI:** 10.3390/diagnostics11112038

**Published:** 2021-11-04

**Authors:** Yu Xie, Julien Oster, Emilien Micard, Bailiang Chen, Ioannis K. Douros, Liang Liao, François Zhu, Marc Soudant, Jacques Felblinger, Francis Guillemin, Gabriela Hossu, Serge Bracard, on behalf of the THRACE Investigators

**Affiliations:** 1 IADI, Université De Lorraine, INSERM, F-54000 Nancy, France; zn000209@whu.edu.cn (Y.X.); julien.oster@inserm.fr (J.O.); bailiang.chen@inserm.fr (B.C.); ioannis.douros@loria.fr (I.K.D.); l.liao@chru-nancy.fr (L.L.); f.zhu@chu-nancy.fr (F.Z.); j.felblinger@chru-nancy.fr (J.F.); g.hossu@chru-nancy.fr (G.H.); 2 Department of Neurology, Zhongnan Hospital of Wuhan University, Wuhan 430000, China; 3 CIC, Innovation Technologique, Université de Lorraine, Inserm, CHRU-Nancy, F-54000 Nancy, France; e.micard@chru-nancy.fr; 4 Université de Lorraine, CNRS, Inria, LORIA, F-54000 Nancy, France; 5 Department of Diagnostic and Interventional Neuroradiology, CHRU Nancy, F-54000 Nancy, France; 6 CIC, Epidémiologie Clinique, Université de Lorraine, Inserm, CHRU-Nancy, F-54000 Nancy, France; m.soudant@chru-nancy.fr (M.S.); francis.guillemin@chru-nancy.fr (F.G.)

**Keywords:** stroke, ischemia, outcome, thrombectomy

## Abstract

Pretreatment ischemic location may be an important determinant for functional outcome prediction in acute ischemic stroke. In total, 143 anterior circulation ischemic stroke patients in the THRACE study were included. Ischemic lesions were semi-automatically segmented on pretreatment diffusion-weighted imaging and registered on brain atlases. The percentage of ischemic tissue in each atlas-segmented region was calculated. Statistical models with logistic regression and support vector machine were built to analyze the predictors of functional outcome. The investigated parameters included: age, baseline National Institutes of Health Stroke Scale score, and lesional volume (three-parameter model), together with the ischemic percentage in each atlas-segmented region (four-parameter model). The support vector machine with radial basis functions outperformed logistic regression in prediction accuracy. The support vector machine three-parameter model demonstrated an area under the curve of 0.77, while the four-parameter model achieved a higher area under the curve (0.82). Regions with marked impacts on outcome prediction were the uncinate fasciculus, postcentral gyrus, putamen, middle occipital gyrus, supramarginal gyrus, and posterior corona radiata in the left hemisphere; and the uncinate fasciculus, paracentral lobule, temporal pole, hippocampus, inferior occipital gyrus, middle temporal gyrus, pallidum, and anterior limb of the internal capsule in the right hemisphere. In conclusion, pretreatment ischemic location provided significant prognostic information for functional outcome in ischemic stroke.

## 1. Introduction

An accurate prediction of functional outcome at the acute stage of ischemic stroke is important for personalized treatment decision-making and early rehabilitation strategies [1]. Mechanical thrombectomy has been proven to be beneficial in acute ischemic stroke patients with large vessel occlusions and has been widely used in the clinical setting [2,3,4,5,6,7,8,9]. It is of great clinical interest to investigate the pretreatment profile in patients appropriate for mechanical thrombectomy.

Several clinical and imaging features have been proposed as appropriate candidates to predict post-stroke functional outcome. The most robust are age, initial stroke severity evaluated by the National Institutes of Health Stroke Scale (NIHSS) score, and infarct volume [10,11,12]. However, these factors are still not sufficient in predicting outcome [13,14]. Infarct location on post-treatment imaging has been proposed as an additional prognostic factor for stroke outcome [15,16,17,18]. However, there is little evidence about the relationship between pretreatment ischemic location and outcome, probably because the lesion progresses rapidly in the acute stage of stroke onset.

In this study, we investigated whether the pretreatment (within 4.5 h of stroke onset) ischemic location could provide additional prediction value for functional outcome at 3 months after mechanical thrombectomy in anterior circulation ischemic stroke. The ischemic location was analyzed at both the hemispheric scale and the atlas-based regional scale. Logistic regression and support vector machine (SVM) techniques were used in the regional scale investigation [19,20].

## 2. Materials and Methods

### 2.1. Patient Selection and Clinical Assessment

We analyzed patients in the THRACE (Thrombectomie des Artères Cérébrales) study (Clinical Trial Registration-URL: http://www.clinicaltrials.gov. Unique identifier: NCT01062698 (accessed on 30 October 2019)), a randomized controlled multicenter trial in France between 2009 and 2015, aimed at comparing intravenous thrombolysis plus mechanical thrombectomy (IVTMT) and intravenous thrombolysis alone (IVT) in patients with acute ischemic stroke due to proximal arterial occlusion, aged 18–80 years, and with an NIHSS score of 10 to 25. The study design and patient inclusion criteria have been described in detail previously [17]. The study protocol was approved by the Comité de Protection des Personnes Est III Ethics Committee and the research boards of the participating centers. All patients or their legal representatives provided written informed consent. In the present study, we selected patients assigned to the IVTMT group who had an anterior circulation occlusion and pretreatment diffusion-weighted imaging (DWI) data.

Demographic and clinical data and functional outcome were extracted from the THRACE database. Functional outcome at 3 months was measured using the modified Rankin Scale (mRS), which consists of 7 grades, from 0 (no symptoms) to 6 (death). The mRS was dichotomized into 0–2 versus 3–6, with 0–2 indicating functional independence and 3-6 indicating poor functional outcome.

We excluded patients with incomplete images or with severe atrophy or hydrocephaly as they could result in inaccurate ischemic location assessment. Patients without a baseline NIHSS score or follow-up mRS were also excluded.

### 2.2. Image Acquisition, Segmentation, and Normalization

Pretreatment DWI data were acquired on 1.5T or 3T MRI scanners within 4.5 h of stroke onset (General Electric Healthcare, WI; Philips medical systems, Best, The Netherlands; Siemens, Erlangen, Germany) according to the established local routines.

Ischemic lesions were segmented semi-automatically with in-house developed software after applying a threshold of the apparent diffusion coefficient <615 × 10^−6^ mm^2^/s on apparent diffusion coefficient maps [21]. The generated lesion masks were then normalized to standard Montreal Neurological Institute space using statistical parametric mapping software (SPM12, Wellcome Trust Centre for Neuroimaging, London, UK). All the image post-processing results were checked by a junior neurologist (<5 years’ experience of reading stroke images) and verified again by an experienced neuroradiologist (>5 years’ experience of reading stroke images), both blind to clinical information.

### 2.3. Regional Ischemic Location Determination

Regional ischemic location was determined according to the Automated Anatomical Labeling (AAL) Atlas (as gray matter atlas) and the Johns Hopkins University White Matter Labels (1 mm) (JHU-WM) Atlas (for white matter tracks) available in the MRIcron software package (http://people.cas.sc.edu/rorden/mricron/index.html (version released on August 2014) (accessed on 30 October 2019)) [22]. The determination of ischemic location was performed in MATLAB (https://www.mathworks.com/products/matlab.html (accessed on 30 October 2019), Mathworks Inc., Sherborn, MA, USA). The ischemic percentage in each anatomical region was calculated.

### 2.4. Prediction Model Building

Two machine learning approaches, logistic regression (linear model) and SVM with radial basis functions (non-linear model), were used to build prediction models. These two approaches were first tested using only conventional parameters (age, baseline NIHSS score, and lesional volume) to build 3-parameter models (LogReg-M_p3_ model and SVM-M_p3_ model). The M_p3_ prediction models were then retrained by including the ischemic percentage in each brain region and by automatically selecting the best subset of parameters using either a least absolute shrinkage and selection operator (LASSO) regularization for the logistic regression or a feature ranking method for the SVM (4-parameter models: LogReg-M_p4_ model and SVM-M_p4_ model) [23,24]. The F_1_ score (a measure of a test’s accuracy, ranges from a best value of 1 and worst value of 0) was used to measure the impact of adding features to the SVM model on prediction performance. To assess the generalizability of our models, a repeated cross-validation was performed for each model, which means that the dataset was divided into 10 subsets. The models were then trained on 9 subsets (“training” set) and tested on the remaining subset (or “validation” set). This process was repeated to use all 10 subsets for evaluation to get one accuracy score. The random division of subsets was repeated 100 times, thus providing 100 accuracy scores for each model. All reported performance scores were obtained on the validation subsets.

### 2.5. Statistical Analysis

Baseline characteristics and functional outcome were compared between left- and right-hemisphere stroke patients. Continuous variables were tested for normality using the Shapiro–Wilk test and described in the form of the mean and standard deviation (SD) if normally distributed, or in the form of the median and interquartile range (IQR) if not. Categorical variables were expressed as proportions. Two-sided comparisons were performed by Wilcoxon test and chi-square test, respectively. The assessment of the difference between the accuracy scores using different prediction models was evaluated by Wilcoxon test. Further, α = 0.05 was chosen as the significance level.

**Figure 1:**
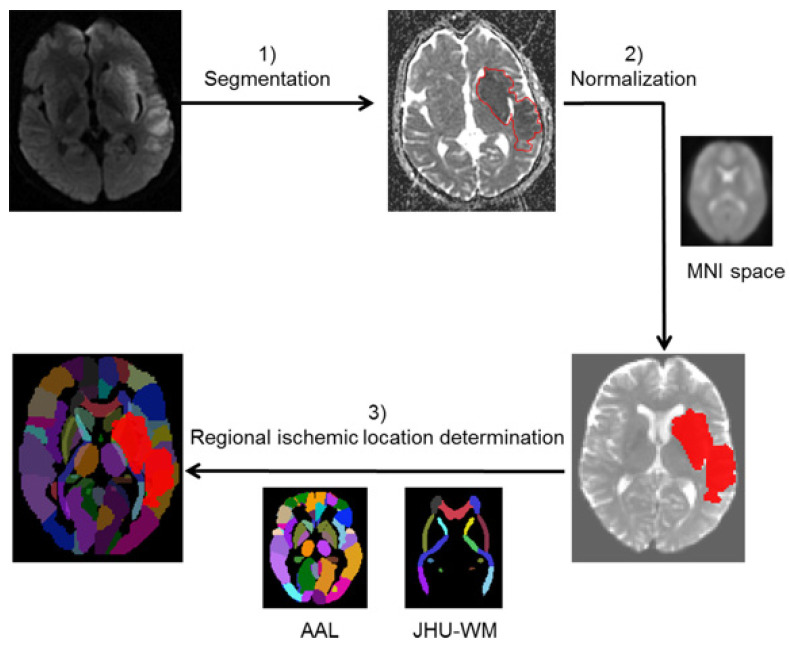
Workflow of lesion location determination: (1) Ischemic lesions were segmented semi-automatically. (2) The generated lesion masks were then normalized to standard Montreal Neurological Institute space using SPM12. (3) The regional ischemic location was determined according to Automated Anatomical Labeling Atlas and the Johns Hopkins University White Matter Labels (1 mm) Atlas. Abbreviations: MNI = Montreal Neurological Institute; AAL = Automated Anatomical Labeling Atlas; JHU-WM = Johns Hopkins University White Matter Labels (1 mm) Atlas.

The data analysis process is presented in Figure 1. The whole workflow was automated and can be processed in real time (about 2 min on a workstation with Intel Xeon W3680-3.3 GHz 6 core CPU). All the statistical analyses were performed using R statistical software (version 3.3.2; R Foundation for Statistical Computing, Vienna, Austria) and MATLAB (https://www.mathworks.com/products/matlab.html (accessed on 30 October 2019), Mathworks Inc., Sherborn, MA, USA). The LIBSVM package was used for the implementation of SVM models [25].

## 3. Results

### 3.1. Patient Characteristics

**Figure 2:**
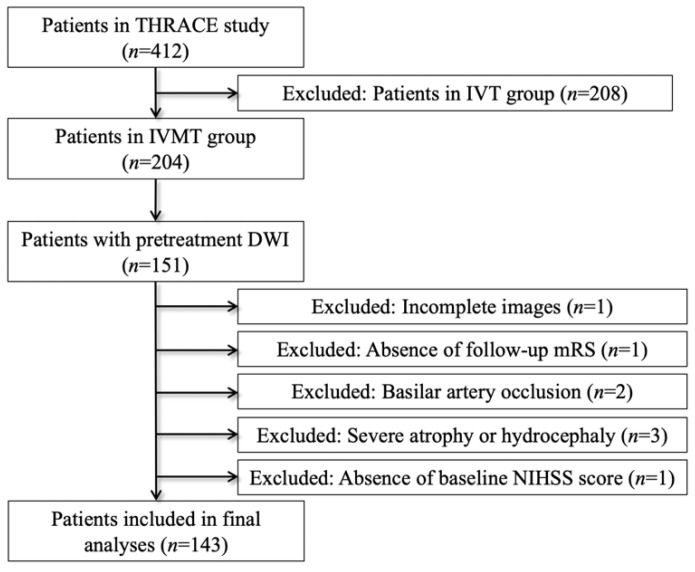
Patient selection flowchart. Abbreviations: IVT = intravenous thrombolysis; IVTMT = intravenous thrombolysis plus mechanical thrombectomy; DWI = diffusion-weighted imaging; mRS = modified Rankin Scale.

A total of 143 acute stroke patients (59 female and 84 male) met the inclusion criteria for our study (Figure 2). The cohort had a median age of 66 years (IQR 54–74). Median ischemic volume was 20.45 mL (IQR 10.28–50.97). Seventy-four (51.75%) patients had left-hemisphere stroke.

### 3.2. Effect of Hemispheric Lateralization on Functional Outcome

The baseline characteristics and functional outcome of the left- and right-hemisphere stroke patients are listed in Table 1. The baseline NIHSS score was significantly higher in the left-hemisphere stroke patients than in the right-hemispheric patients (*p* < 0.001). Functional outcome at 3 months was comparable for stroke in either hemisphere.

### 3.3. Effect of Ischemic Topography on Functional Outcome

**Figure 3:**
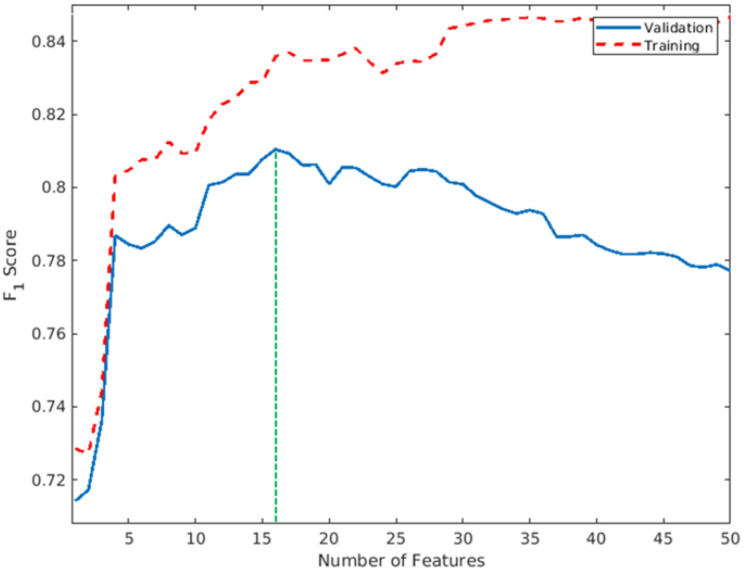
F_1_ accessed on 30 October 2019 score obtained on the average training (red dotted line) and validation (blue line) sets during cross-validation performed by SVM after adding different features. The model reached the highest performance on the validation set when using 16 features (green dotted line). Abbreviation: SVM = support vector machine.

Table 2 assembles the accuracy, sensitivity, specificity, positive predictive value (PPV), negative predictive value (NPV), and the area under the curve (AUC) of the four different models. A linear model using only conventional parameters (LogReg-M_p3_ model) reached an AUC of 0.77, which was similar to the nonlinear model (SVM-M_p3_ model). Adding the ischemic percentage in each brain region led to an increased AUC, with 0.78 (increment of 0.01) for the linear model (LogReg-M_p4_ model) and 0.82 (increment of 0.05) for the nonlinear model (SVM-M_p4_ model). The AUC of the SVM-M_p4_ model was significantly higher than that of the LogReg-M_p4_ model (*p* < 0.001). It can also be noted that the lesional volume was not selected as a predictor by the automated approach in the SVM-M_p4_ model. As shown in Figure 3, the model reached the highest performance on the validation set when using 16 features. Regions with a marked impact on functional outcome prediction and their function included the uncinate fasciculus, postcentral gyrus, putamen, middle occipital gyrus, supramarginal gyrus, and posterior corona radiata in the left hemisphere; and the uncinate fasciculus, paracentral lobule, temporal pole, hippocampus, inferior occipital gyrus, middle temporal gyrus, pallidum, and anterior limb of the internal capsule in the right hemisphere.

## 4. Discussion

To the best of our knowledge, our work is the first to attempt to identify the additional prognostic value of pretreatment ischemic location for functional outcome, compared to the conventional parameters (age, baseline NIHSS score, and lesional volume). We investigated the impact of both hemispheric lateralization (right or left) and atlas-based regional location on functional outcome. Furthermore, a nonlinear machine learning method was utilized in our study as such an approach can allow a better classification than using linear models, especially when incorporating additional features (percentage of ischemic tissue in each brain region).

At the hemispheric scale, our results suggest that the lesional hemispheric lateralization does not significantly impact on functional outcome, in line with a previous study [26]. The baseline NIHSS score in right-hemisphere stroke patients was significantly lower than that in left-sided stroke patients, indicating that the NIHSS score placed greater emphasis on left-hemisphere dysfunction, in line with previous research [27]. Therefore, ischemic lateralization should be taken into consideration when evaluating stroke severity.

At the atlas-based regional scale, ischemic location was demonstrated to be able to provide additional prediction value for functional outcome. Fourteen regions, which are involved in cognitive function, motor and sensory function, and visual function, are demonstrated to play important roles in outcome prediction.

The uncinate fasciculus is involved in memory, language, social-emotional processing tasks, and executive function [28,29,30]. The high predictive value of the uncinate fasciculus on functional outcome is in accordance with the findings reported previously [16]. The right temporal lobe is reported to be implicated in semantic dementia, behavior, and socioemotional functioning [31]. The hippocampus is essential for the storage of long-term memory. The anterior limb of the internal capsule is an important thalamo-frontal white matter tract and is reported to be related to depression [32]. These regions were also shown to have a high predictive value in other studies on functional outcome [15,16,17]. We infer that lesions in the above-mentioned regions could impact post-stroke rehabilitation, thereby influencing functional outcome at 3 months.

The putamen is a part of the striatum, which receives input from cortical areas and thalamic nuclei and sends signal to other components of the basal ganglia. The pallidum is an output nucleus of the basal ganglia. Both the putamen and the pallidum participate in the direct and indirect pathways of movement, and their impairment could cause hypokinetic or hyperkinetic disorders [33]. The corona radiata is a part of the corticospinal tract. Our findings are consistent with previous studies that suggested the importance of motor pathways in stroke functional outcome prediction [15,16,17].

The postcentral gyrus exerts a critical role in the initiation and execution of purposive movements [34]. The paracentral lobule, which connects the medial portion of the precentral and postcentral gyri, controls motor and sensory innervation. The supramarginal gyrus is a part of the somatosensory association cortex and plays a critical role in ideomotor transformation: the integration of conceptual knowledge and motor representations into meaningful actions [35]. It could be inferred that damage to the somatosensory cortex disturbs the kinesthetic input conveying to motor areas and therefore impacts the functional outcome. This result is in line with a previous study, which supported a critical role of somatosensory cortices in motor skill recovery [36].

Visual feedback could partly substitute for somatosensory information and thus compensate for the motor task deficit [34]. This could explain why regions in the occipital lobe, where the visual processing center is located, were involved in functional outcome prediction in our study.

However, certain regions implicated in poor outcome prediction in previous studies did not present marked predictive value in our study, such as the external capsule, the insula, and the left superior temporal gyrus [15,16,17]. This may be due to the fact that ischemic location was evaluated within 4.5 h after stroke onset in our study, while the other studies performed imaging on day 2 or 3. The dynamic process of ischemia, especially at the hyperacute phase, could result in lesion location evolution.

The mRS has been considered as a functional disability assessment tool, which mainly emphasizes motor recovery [37]. Our findings, however, demonstrated that brain regions associated with the mRS score were involved in various aspects of neurological functionalities. This could be explained by the fact that nonphysical functions (such as somatosensory, cognition, and post-stroke mood disturbance) would impact the rehabilitation process, thus further affecting functional outcome at 3 months [38].

Our results also shed new light on the application of machine learning methods in stroke outcome prediction. Machine learning approaches have already been proposed for stroke outcome prediction using different parameters, such as baseline NIHSS score, intracerebral hemorrhage transformation, recanalization success, and age, which obtained promising accuracy (approaching 70%) [39]. Our work improved the prediction capacity after adding the lesional location to the model.

Our study has several limitations. We tried to recruit homogeneous patients without bias related to treatment, which gave rise to a relatively small sample size. Due to the lack of an age-matched atlas, we applied young adult-derived atlases to elderly patients. As this might cause potential bias, we checked all the normalized images and excluded any that were problematic. Information on handedness, which influences functional lateralization, was unavailable in our cohort. Different MRI scanners were used in our study, which may have influenced the results. Although the performance of our technique is convincing, with an AUC of 0.82, it might be inadequate to make a treatment decision, and a more comprehensive model with other factors should be further investigated, such as the ASPECT score, successful recanalization, stroke onset to needle time, etc. Furthermore, the origin of the stroke was not collected during the THRCAE study. It was therefore difficult to include this parameter in our models and study its influence on the functional outcome, although a recent study has suggested it could impact the outcome [40]. Finally, the proposed technique should be further evaluated on an independent external dataset during a future study and needs to be validated during a randomized clinical trial before being transferred into clinical routine.

## 5. Conclusions

Our study suggests that pretreatment ischemic location could provide important prognostic value for functional outcome in acute anterior circulation stroke, in addition to age, the baseline NIHSS score, and ischemic volume. Moreover, an advanced model using the SVM technique was developed for multifactorial stroke outcome prediction.

## Figures and Tables

**Table 1:** Baseline characteristics and functional outcome of left- vs. right-hemisphere stroke patients.

	Left Hemisphere (*n* = 74)	Right Hemisphere (*n* = 69)	*p*
Age, years	64 (52–74)	68 (57–73)	0.34
Sex, female/male	31/43	28/41	0.99
Diabetes mellitus	4/73 (5.48%)	4/68 (5.88%)	0.99
Hypertension	33/74 (44.59%)	33/67 (49.25%)	0.70
Smoking (current or past)	36/63 (57.14%)	23/64 (35.94%)	0.03 *
Hypercholesterolemia	34/62 (54.84%)	26/63 (41.27%)	0.18
Coronary disease	7/70 (10.00%)	10/67 (14.93%)	0.54
History of stroke	4/71 (5.63%)	4/65 (6.15%)	0.99
Admission glucose, g/L	1.2 (1.0–1.5)	1.2 (1.0–1.4)	0.62
Baseline NIHSS score	20 (18–22)	15 (12–17)	0.001 *
Time from stroke onset to imaging, min	113 (85–143)	116 (93–127)	0.65
Time from stroke onset to randomization, min	173 (143–202)	161 (142–183)	0.13
Occlusion location, ICA: M1	10:64	5:64	0.34
ASPECT score	7 (5–8)	7 (6–8)	0.04 *
Fazekas score	0 (0–1)	1 (0–1)	0.10
Functional outcome (mRS)	2 (1–4)	2 (1–4)	0.33
Favorable functional outcome (mRS ≤ 2)	40/74 (54.05%)	42/69 (60.87%)	0.51
Ischemic volume, mL	21.58 (11.14–54.39)	19.20 (10.24–32.65)	0.51

Quantitative data are presented as median (interquartile range); qualitative data are presented as no. (%). NIHSS = National Institutes of Health Stroke Scale; ICA = intracranial internal carotid artery; M1 = proximal portion of middle cerebral artery; ASPECT = Alberta Stroke Program Early CT Score; mRS = modified Rankin Scale. * = *p* value < 0.05.

**Table 2:** Model performances using repeated cross-validation.

Model	LogReg-M_p3_	LogReg-M_p4_	SVM-M_p3_	SVM-M_p4_
Accuracy (Mean ± SD)	0.70 ± 0.01	0.73 ± 0.01	0.71 ± 0.02	0.77 ± 0.01
Sensitivity (Mean ± SD)	0.60 ± 0.01	0.64 ± 0.02	0.60 ± 0.03	0.66 ± 0.03
PPV (Mean ± SD)	0.66 ± 0.02	0.71 ± 0.02	0.68 ± 0.02	0.77 ± 0.02
NPV (Mean ± SD)	0.72 ± 0.01	0.75 ± 0.01	0.72 ± 0.02	0.77 ± 0.01
AUC (Mean ± SD)	0.77 ± 0.01	0.78 ± 0.01	0.77 ± 0.01	0.82 ± 0.01

Age, baseline National Institutes of Health Stroke Scale score, and lesional volume were included in 3-parameter models (LogReg-M_p3_ and SVM-M_p3_) for outcome prediction. Ischemic percentage in each brain region was also included in 4-parameter models (LogReg-M_p4_ and SVM-M_p4_). PPV = positive predictive value. NPV = negative predictive value. AUC = area under the curve. LogReg = logistic regression; SVM = support vector machine; SD = standard deviation.

## Data Availability

The data/code that support the findings of this study are available from the corresponding author (S.B.), upon reasonable request.

## Supplementary Materials

The following are available online at https://www.mdpi.com/article/10.3390/diagnostics11112038/s1.
