# Supplementary material for: Impact of Pretreatment Ischemic Location on Functional Outcome after Thrombectomy
Source: Diagnostics (Basel). 2021 Nov 4;11(11):2038. doi: 10.3390/diagnostics11112038 (PMC8625281; doi:10.3390/diagnostics11112038)
Supplement: Supplementary file 1 [file diagnostics-11-02038-s001.zip › diagnostics-1393396-Supplementary.pdf]

List of THRACE, Investigators and affiliations :

CHU Nancy: Xavier Ducrocq, René Anxionnat, Pierre-Alexandre Baillot, Charlotte Barbier, Anne-Laure Derelle, Jean-Christophe Lacour, Sébastien Richard. ·

Paris CHU Pitié- Salpétrière: Yves Samson, Nader Sourour, Flore Baronnet-Chauvet, Frédéric Clarencon, Sophie Crozier, Sandrine Deltour, Federico Di Maria, Raphael Le Bouc, Anne Leger, Gurkan Mutlu, Charlotte Rosso, Zoltan Szatmary, Marion Yger, Chiara Zavanone,.

CHU Reims : Serge Bakchine, Laurent Pierot, Nathalie Caucheteux, Laurent Estrade, Krzysztof Kadziolka, Alexandre Leautaud, Céline Renkes, Isabelle Serre.

CHU Nantes : Hubert Desal, Benoît Guillon, Claire Boutoleau-Bretonniere, Benjamin · Daumas-Duport, Solène De Gaalon, Pascal Derkinderen, Sarah Evain, Fanny Herisson, David-Axel Laplaud ,Thibaud Lebouvier, Alina Lintia-Gaultier, Hélène Pouclet-Courtemanche, Tiphaine Rouaud, Violaine Rouaud Jaffrenou, Aurélia Schunck, Mathieu Sevin, Allouet, Frederique Toulgoat, Sandrine Wiertlewski.

CHU Rennes : Jean-Yves· Gauvrit , Thomas Ronziere, Vincent Cahagne, Jean-Christophe Ferre, Jean-François· Pinel.

Paris-Ste Anne: Jean-Louis· Mas, Jean-François Meder, Amen-Adam Al Najjar-Carpentier, Julia Birchenall, Eric Bodiguel, David Calvet, Valérie Domigo, Sylvie Godon-Hardy, Vincent Guiraud, Catherine Lamy, Loubna Majhadi, Ludovic Morin, Olivier Naggara, Denis Trystram, Guillaume Turc.

CHU Bordeaux: Jérôme Berge, Igor Sibon, Patrice Menegon, Xavier Barreau, François Rouanet, Sabrina Debruxelles ,Annabelle Kazadi, Pauline Renou, Olivier Fleury.

CHU Angers: Anne Pasco-Papon, Frédéric Dubas, Jildaz Caroff, Sophie Godard Ducceschi, Marie-Aurélie Hamon, Alderic Lecluse, Guillaume Marc.

CHU Dijon: Maurice Giroud, Frédéric Ricolfi, Yannick Bejot, Adrien Chavent ,Arnaud Gentil, Apolline Kazemi, Guy-Victor· Osseby Charlotte Voguet.

CHU Nice : Marie-Hélène Mahagne Jacques Sedat, Yves Chau, Laurent Suissa, Sylvain Lachaud. Paris

CHU Lariboisière: Emmanuel Houdart, Christian Stapf, Frédérique Buffon, Porcher Hugues Chabriat, Pierre Guedin, Dominique Herve, Eric Jouvent, Jérôme Mawet, Jean-Pierre· Saint-Maurice, Hans-Martin Schneble,

CHU Lyon: Francis Turjman, Norbert Nighoghossian ,Nadia-Nawel Berhoune, Françoise Bouhour, Taa-Hee Cho Laurent Derex, Sandra Felix, Hélène Gervais-Bernard, Benjamin Gory, Luis Manera, Laura Mechtouff, Thomas Ritzenthaler ,Roberto Riva ,Fabrizio Salaris, Silvio · Caroline Tilikete.

Paris -Fondation Rothschild : Raphael Blanc, Michaël Obadia, Mario Bruno Bartolini, Antoine Gueguen, Michel Piotin, Silvia Pistocchi, Hocine Redjem.

CHU Poitiers: Jacques Drouineau, Jean-Philippe· Neau, Gaelle Godeneche, Matthias Lamy, Emilia Marsac, Stephane Velasco.

CHU Clermont-Ferrand: Pierre Clavelou, Emmanuel Chabert, Nathalie Bourgois, Catherine Cornut-Chauvinc, Anna Ferrier, Jean Gabrillargues, Betty Jean, Anna-Raquel· Marques, Nicolas Vitello.

CHU Grenoble: Olivier Detante, Marianne Barbieux, Kamel Boubagra, Isabelle Favre, Wiki Katia Garambois, Florence Tahon, Vasdev Ashok,· Charlotte Voguet.

Hopital Foch. Suresnes: Rodesch Georges, Lapergue Bertrand, Bourdain Frédéric, Evrard Serge, Graveleau Philippe, Decroix Jean Pierre, Wang Adrien, Gueudin Pierre, Oz Coskun.

Hopital Pasteur. Colmar : François Sellal, Guido Ahle, Gabriela Carelli, Marie-Hélène Dugay, Claude Gaultier, Ariel Pablo Lebedinsky, · Lavinia Lita, Raul Mariano Musacchio, Catherine Renglewicz-Destuynder, Alain Tournade, Françis Vuillemet.

CHU Limoges: Francisco Macian Montoro, Charbel Mounayer, Frederic Faugeras, Laetitia Gimenez , Catherine Labach, Géraldine Lautrette. Paris

CHU Bicetre: Christian Denier, Guillaume Saliou, Olivier Chassin, Claire Dussaule, Elsa Melki, Augustin Ozanne, Francesco Puccinelli, Marina Sachet, Sarov Mariana·

CHU Besançon: Jean-François Bonneville, Thierry Moulin, Alessandra Biondi, Elisabeth De Bustos Medeiros, Fabrice Vuillier.

CHU Caen: Patrick Courtheoux, Fausto Viader, Marion Apoil- Brissard, Mathieu Bataille, Anne-Laure Bonnet ,Julien Cogez, Apolline Kazemi ,Emmanuel Touze.

CHU Lille: Xavier Leclerc, Didier Leys, Mohamed Aggour, Pierre Aguettaz, Marie Bodenant, Charlotte Cordonnier, Dominique Deplanque, Marie Girot, Hilde Henon, Erwah Kalsoum, Christian Lucas, Jean-Pierre Pruvo, Paolo Zuniga.

CHU Montpellier: Alain Bonafé, Caroline Arquizan, Vincent Costalat, Paolo Machi, Isabelle Mourand, Carlos Riquelme.

HIA Toulon: Pierre Bounolleau, Charles Arteaga, Anthony Faivre.

CHU Reunion: Marc Bintner, Patrice Tournebize, Cyril Charlin, Françoise Darcel, Pascale Gauthier-Lasalarie, Marcia Jeremenko, Servane Mouton, Jean-Baptiste Zerlauth.
